# Ex vivo expanded tumour-infiltrating lymphocytes from ovarian cancer patients release anti-tumour cytokines in response to autologous primary ovarian cancer cells

**DOI:** 10.1007/s00262-018-2211-3

**Published:** 2018-07-23

**Authors:** Gemma L. Owens, Marcus J. Price, Eleanor J. Cheadle, Robert E. Hawkins, David E. Gilham, Richard J. Edmondson

**Affiliations:** 1 Gynaecological Oncology, Division of Cancer Sciences, School of Medical Sciences, Faculty of Biology, Medicine and Health, Manchester Academic Health Science Centre, University of Manchester, Manchester, UK; 2 St Mary’s Hospital, Central Manchester NHS Foundation Trust, Manchester Academic Health Science Centre, Level 5, Research Floor, Oxford Road, Manchester, M13 9WL UK; 3 Clinical and Experimental Immunotherapy, Division of Cancer Sciences, School of Medical Sciences, Faculty of Biology, Medicine and Health, Manchester Academic Health Science Centre, Manchester Cancer Research Centre, University of Manchester, Wilmslow Road, Manchester, UK; 4 Targeted Therapy Group, Division of Cancer Sciences, School of Medical Sciences, Faculty of Biology, Medicine and Health, Manchester Academic Health Science Centre, Manchester Cancer Research Centre, University of Manchester, Wilmslow Road, Manchester, UK

**Keywords:** Tumour-infiltrating lymphocytes (TIL), T cells, Ovarian cancer, Immunotherapy, Adoptive cell therapy

## Abstract

Epithelial ovarian cancer (EOC) is the leading cause of gynaecological cancer-related death in Europe. Although most patients achieve an initial complete response with first-line treatment, recurrence occurs in more than 80% of cases. Thus, there is a clear unmet need for novel second-line treatments. EOC is frequently infiltrated with T lymphocytes, the presence of which has been shown to be associated with improved clinical outcomes. Adoptive T-cell therapy (ACT) using ex vivo-expanded tumour-infiltrating lymphocytes (TILs) has shown remarkable efficacy in other immunogenic tumours, and may represent a promising therapeutic strategy for EOC. In this preclinical study, we investigated the efficacy of using anti-CD3/anti-CD28 magnetic beads and IL-2 to expand TILs from freshly resected ovarian tumours. TILs were expanded for up to 3 weeks, and then subjected to a rapid-expansion protocol (REP) using irradiated feeder cells. Tumours were collected from 45 patients with EOC and TILs were successfully expanded from 89.7% of biopsies. Expanded CD4^+^ and CD8^+^ subsets demonstrated features associated with memory phenotypes, and had significantly higher expression of key activation and functional markers than unexpanded TILs. Expanded TILs produced anti-tumour cytokines when co-cultured with autologous tumour cells, inferring tumour cytotoxicity. Our findings demonstrate that it is possible to re-activate and expand tumour-reactive T cells from ovarian tumours. This presents a promising immunotherapy that could be used sequentially or in combination with current therapeutic strategies.

**Electronic supplementary material:**

The online version of this article (10.1007/s00262-018-2211-3) contains supplementary material, which is available to authorized users.

## Introduction

Epithelial ovarian cancer has the highest mortality rate of all gynaecological cancers, with more than 4000 deaths each year in the UK [1]. Although many patients with advanced disease will initially achieve a complete response with cytoreductive surgery and platinum-based chemotherapy, recurrence occurs in more than 80% of cases. Efforts to improve survival outcomes are currently focused on the use of dose-dense weekly paclitaxel and the addition of targeted therapies such as anti-angiogenic agents and PARP inhibitors to standard first-line treatment [2–6]. To date, the clinical benefit from these strategies has been marginal and, therefore, there is a clear unmet need for alternative treatment strategies.

There is compelling evidence to suggest epithelial ovarian cancer is immunogenic. Several studies have demonstrated spontaneous anti-tumour immune responses through the identification of tumour-reactive T cells in the peripheral blood, ascitic fluid and within the tumour microenvironment of patients with advanced disease [7–10]. Furthermore, infiltration of CD3^+^ and CD8^+^ T cells into the tumour has been associated with improved overall and progression-free survival in patients with epithelial ovarian cancer [11, 12]. Unfortunately, ovarian tumours also acquire mechanisms to escape immune surveillance, skewing the tumour microenvironment towards a tolerogenic state. These mechanisms include the recruitment of regulatory T cells and MDSCs, expression of co-inhibitory ligands such as PD-L1, reduced antigen presentation and immunosuppressive soluble factors such as TGF-β, IL-10 and IDO [13–17].

Cancer immunotherapy has recently emerged as a clinically efficient tool in several solid tumours. Two PD-1 inhibitors, Pembrolizumab and Nivolumab, have now been approved by NICE for the treatment of metastatic melanoma, and have shown encouraging results in advanced non-small cell lung cancer and renal-cell carcinoma clinical trials [18–20]. Disappointingly, a recent phase II trial of Nivolumab in patients with platinum-resistant disease only noted an objective response in 20% of patients [21]. ACT using endogenous TILs may, therefore, represent an alternative strategy to harness the immune system for therapeutic benefit. In metastatic melanoma, sustained responses have been seen in up to 50% of patients receiving autologous TILs [22]. More recently, TILs with anti-tumour activity have been successfully generated in renal cell, pancreatic and cervical cancers [23–26].

The feasibility of TIL therapy has previously been examined in ovarian cancer, in four early clinical trials ([27–31], reviewed in [32]). The most promising results were reported by Fujita et al. [29], who found that women with advanced ovarian cancer who received adjuvant TILs following debulking surgery and chemotherapy had a 3-year survival of 100% compared to 67.5% in the control group. Although the study suffered from lack of randomization, the results still illustrate the potency of TIL therapy. Despite this, there have been no recently published studies of TIL therapy for epithelial ovarian cancer. In light of recent advances in the understanding of the immunobiology of ovarian cancer and developments in culturing techniques, it is timely to revisit the generation of TILs for the treatment of ovarian cancer. In this pre-clinical study, we developed a protocol that reproducibly generates large numbers of tumour-specific TIL from epithelial ovarian cancer biopsies, suitable for therapeutic use.

## Materials and methods

### Patient samples

Solid tumour biopsies and ascites were collected from patients undergoing surgery for ovarian cancer at St. Mary’s Hospital, Manchester, UK. Solid tumour biopsies were collected into a sterile universal containing MACS Tissue Storage Solution (Miltenyi Biotec, Germany) and transported directly to the laboratory for processing. Ascites was aspirated directly into a FLOVAC liner. Samples were registered and handled in accordance with the Human Tissue Act (2004).

### Tumour disaggregation

Ovarian tumours were dissociated into single-cell suspensions using the MACS human tumour dissociation kit (Miltenyi Biotec, Germany) according to the manufacturer’s protocol. Briefly, tumour biopsies were cut into 2–4 mm^3^ fragments using a sterile scalpel and transferred into a C-tube (Miltenyi Biotec, Germany) with RPMI-1640 (Lonza, Slough, UK) and the appropriate volumes of enzymes H, R and A (Human Tumour Dissociation Kit, Miltenyi Biotec, Germany). The sample was placed onto the GentleMACS dissociator and subjected to three pre-set dissociation programs according to the manufacturer’s instructions. This was interjected by two 30-min incubations at 37 °C under continuous rotation using the MACSmix Tube Rotator (Miltenyi Biotec, Germany). Following disaggregation, the cell suspension was passed through a 100-µm strainer, and centrifuged at 400*g* for 5 min. The supernatant was discarded and the remaining cell pellet was thoroughly resuspended and cells were counted.

### Isolation of cells from ascites

Ascitic fluid samples were centrifuged at 1000*g* for 10 min at 4 °C. The supernatant was discarded, and the cell pellet was resuspended in PBS. The cell suspension was layered onto an equal volume of Lymphoprep, and centrifuged at 1000*g* for 20 min at 4 °C, with the brake off. Following centrifugation, cells were collected from the ascites–Lymphoprep interface and washed with PBS. Cells were separated into a single cell suspension using a 25G needle.

### Initiation of TIL cultures

Initially, cells isolated from tumours/ascites were cultured in 24-well plates at a concentration of 0.5 × 10^6^/ml in T-cell media (RPMI-1640 500 ml supplemented with 10% heat-inactivated FCS, 1% l-glutamine, 100 IU/ml penicillin, 100 µg/ml streptomycin (all Life Technologies, Paisley, UK), 25 mM HEPES and 50 µM β-mercaptoethanol [both from Sigma-Aldrich, Dorset, UK)] with 3000 IU/ml recombinant human IL-2 (Novartis, UK). TILs were mitogenically stimulated by the addition of Dynabeads^®^ Human T-Activator CD3/CD28 (Life Technologies, Paisley, UK) at 1:1 bead to T-cell ratio. Cells were incubated at 37 °C, 5% CO_2_, 95% humidity. On alternate days, half of the media was removed and replaced with fresh media supplemented with 3000 IU/ml of IL-2.

After 7 days in culture, the Dynabeads^®^ were removed using a magnet (Invitrogen, Norway). TILs were counted and reseeded at 1 × 10^6^ cells/ml in T-cell media supplemented with 1000 IU/ml of IL-2. TILs were counted and reseeded in fresh T-cell media and IL-2 on alternate days, to maintain a density of 1 × 10^6^ cells/ml. Cultures were maintained for a maximum of 20 days, at which time functional assays were performed.

### Small-scale rapid expansion protocol (REP)

1 × 10^5^ frozen TILs were incubated with feeder cells consisting of irradiated (30 Gy) PBMCs mixed from three healthy donors at a 200:1 feeder cell to TIL ratio. Cells were cultured at a concentration of 1 × 10^6^ cells/ml with 30 ng/ml anti-CD3 antibody (OKT3, OrthoBiotech, High Wycombe, UK) and 3000 IU/ml of IL-2. Fresh IL-2 was added on alternate days and reduced to 1000 IU/ml at day 7. TILs were rapidly expanded for 14 days.

### Primary culture

Autologous primary ovarian cancer cultures were initiated in parallel with the TIL cultures. Briefly, tumour samples were dissected into approximately 3 mm^3^ fragments and transferred to T25 flasks containing collagenase/dispase (Roche, UK) at a concentration of 1 mg/1 ml in RPMI-1640 supplemented with 20% FCS. The tumour cells were incubated for 2 h at 37 °C on a shaker. Following disaggregation, the cell suspension was transferred to a 50-ml Falcon and centrifuged at 400*g* for 5 min. The supernatant was discarded and the cell pellet was washed with PBS and was resuspended in complete media (RPMI 1640, 20% FCS, 20 mM l-glutamine, 1% penicillin and streptomycin) and placed in a T75 flask. Primary cultures were subcultured when they reached approximately 80% confluency. To ensure that the primary cultures represented epithelial ovarian cancer cells, they were characterized as described previously by O’Donnell et al. [33].

### IFNγ ELISA

Expanded TILs and autologous tumour cells were co-cultured at an E:T ratio of 1:1 (1 × 10^5^ target cells). Simultaneously, TILs were cultured alone (negative control) and with 50 ng/ml PMA and 1 µg/ml Ionomycin (positive control). The cultures were incubated at 37 °C, 5% CO_2_, 95% humidity for 24 h, before the supernatant was harvested. Supernatants were analysed for IFNγ production using the IFNγ ELISA kit (Diaclone, France) according to the manufacturer’s instructions. Where MHC class I and class II blocking experiments were performed, tumour cells were incubated with 50 µg/ml of anti-HLA ABC (clone W6/32, Biolegend, UK) and/or anti-HLA DR DP DQ (clone Tu39, BD Biosciences, UK) for 45 min at 4 °C prior to setting up the co-culture.

### Flow cytometry

Phenotype of TILs was studied on the cells from the tumour digest and following expansion. Supplementary table 1 lists all flow cytometry antibodies used (all mouse anti-human). FcR block (Miltenyi Biotec, Germany) was used prior to staining. Intracellular staining for FoxP3, IFNγ, IL-2 and TNFa was carried out using the Fix-Permeabilisation Kit in accordance with the manufacturer’s protocol (eBioscience). Data were acquired on a FACS Calibur 4-colour flow cytometer or LSR-Fortessa (BD Biosciences, UK). Data were analysed using FlowJo v. 7.6.2 software (Tree Star Inc, Ashland, OR).

### Molecular classification of the tumour samples

Total RNA was extracted from fresh frozen tumour samples using the Qiagen RNeasy kit (Qiagen, Valencia, CA, USA) according to the manufacturer’s instructions. RNA yield was measured using a NanoDrop Spectrophotometer (NanoDrop Technologies Inc.) and RNA integrity assessed using the Agilent 4200 TapeStation system (Agilent Technologies). Expression levels of the subtype-specific genes (48 genes) were assessed using the Nanostring platform. Tumours were then classified into four distinct molecular subtypes (C1, C2, C4, C5) as previously described by Leong et al. [34]. Briefly, samples from the Tothill et al. [35] paper were used as the learning set to develop a classification model. This model was then used to predict the subtypes of the samples in our cohort. This classification algorithm provides the probability of the subtype of every sample and the sample is given the label with the maximum probability.

### Statistical analysis

Statistical analyses were performed using GraphPad Prism (Version 7.0, GraphPad Software Inc., La Jolla, CA).

## Results

### Ovarian TILs can be readily expanded

Although TILs can readily be expanded from melanoma biopsies by the addition of high-dose IL-2 alone [36], expanding TILs from other solid tumours has been significantly more challenging. To determine the feasibility of isolating and expanding TILs from epithelial ovarian cancers, we adapted our previous experience with renal cell carcinoma to establish ovarian TIL cultures [23]. Solid tumour biopsies and/or ascitic fluid were collected from 45 patients undergoing surgery for an epithelial ovarian cancer at St. Mary’s Hospital, Manchester. Tumour characteristics and patient demographics are outlined in Table 1. The vast majority of the tumours collected were of high-grade serous type (*n* = 40), which reflects the predominance of this subtype in clinical practice. All patients who underwent interval debulking surgery (IDS) received three cycles of neoadjuvant carboplatin and paclitaxel.

**Table 1:** Clinical characteristics of patients

Characteristics	*N* = 45
Age (years), median + range	64.5 ± 10.5
Histological subtype, *n* (%)	
High-grade serous	40 (88.9%)
Low-grade serous	2 (4.4%)
Clear cell carcinoma	2 (4.4%)
Carcinosarcoma	1 (2.2%)
FIGO stage, *n* (%)	
FIGO I	1 (2.2%)
FIGO II	1 (2.2%)
FIGO III/IV	43 (95.6%)
Type of surgery, *n* (%)	
Primary surgery	26 (57.8%)
Interval debulking surgery	16 (35.6%)
Recurrent disease	3 (6.7%)

In total, 68 samples representing solid biopsies and ascites were collected from 45 patients, which included multiple biopsies from 5 patients with disseminated disease, to compare TIL expansion and phenotype between different anatomical sites. Most biopsies were from omental metastases (*n* = 37) or the primary ovarian tumour (*n* = 12). Other sites included the liver, peritoneum, diaphragm and the uterus.

**Fig. 1:**
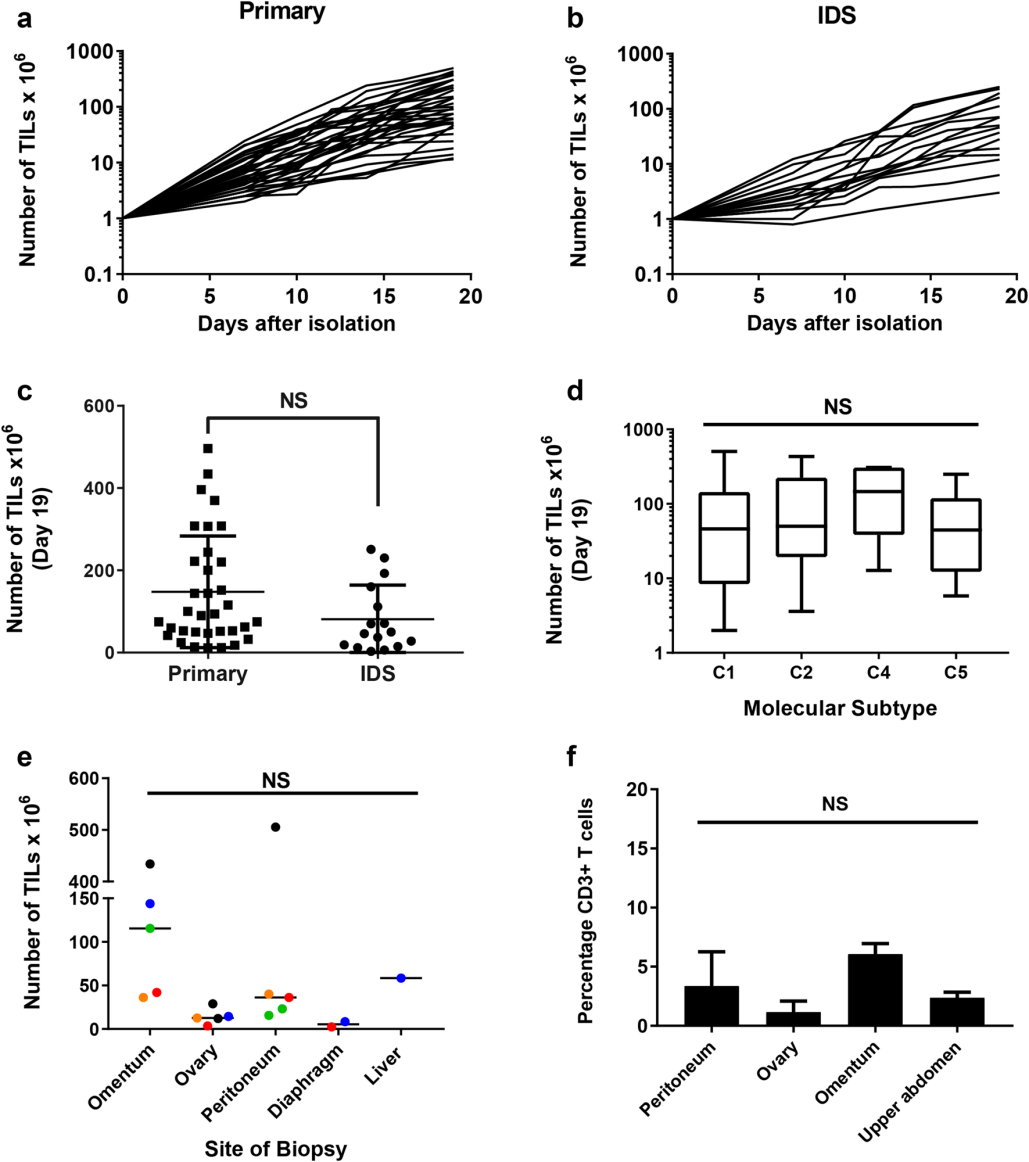
Expansion of TILs from epithelial ovarian cancer biopsies. **a** Growth profiles of TILs during the 19-day initial culture period, isolated from patients undergoing primary surgery or **b** interval debulking surgery (IDS) following neo-adjuvant chemotherapy. Data are shown on a logarithmic scale. **c** Comparison of the number of TILs expanded from biopsies taken at primary surgery and IDS. Mean and SD are shown. *p* < 0.05, *NS* not significant, Student’s *t* test. **d** Comparison of the number of TILs expanded from the four distinct molecular subtypes of ovarian cancer (C1—mesenchymal; C2—immune; C4—differentiated; C5—proliferative) [35]. Data represent the median, upper and lower quartiles and range. **e** Comparison of number of TILs expanded from biopsies taken from different anatomical sites of disease from five patients. Colours denote individual patients; one-way Anova. **f** Percentage of CD3 T cells in the tumour disaggregates from different anatomical sites; one-way Anova

Following tumour disaggregation using GentleMACS, we initiated TIL cultures by mitogenically stimulating 4 × 10^6^ cells with anti-CD3/anti-CD28 beads and high-dose IL-2. TILs were successfully expanded to a minimum of 1 × 10^7^ cells at day 19, from 61 of the 68 (89.7%) biopsies (Fig. 1a, b). Final TIL counts at day 19 varied considerably and ranged from 1.2 to 50.5 × 10^7^ cells. Interestingly, we observed no difference in the average number of TILs expanded from biopsies taken from treatment naïve patients and those who had received neoadjuvant chemotherapy (Fig. 1c). Three of the patients in our cohort had surgery for recurrent disease. We were unable to expand TIL from two of these patients. There was no correlation between efficiency of TIL expansion and the molecular subtype of ovarian cancer as determined by Nanostring (Fig. 1d).

To assess the impact of tumour heterogeneity on the distribution and functional activity of TILs, we isolated TILs from multiple biopsies from different anatomical sites of disease in five patients. TILs were successfully expanded from all anatomical sites in four of the five patients. We observed differences in the final TIL yield from different anatomical sites within the same patient (Fig. 1e). This was most marked in patient 47, where the final cell counts ranged from 1.2 to 50.5 × 10^7^ in the cultures initiated from the left ovary and the peritoneum, respectively. In general, TILs isolated from omental biopsies appeared to expand more readily than TILs isolated from other tumour sites although this was not statistically significant (*p* = 0.55). Importantly, there was no difference between the percentages of CD3 T cells in the tumour disaggregate from different sites (Fig. 1f).

### Ovarian TILs have memory phenotypes

**Fig. 2:**
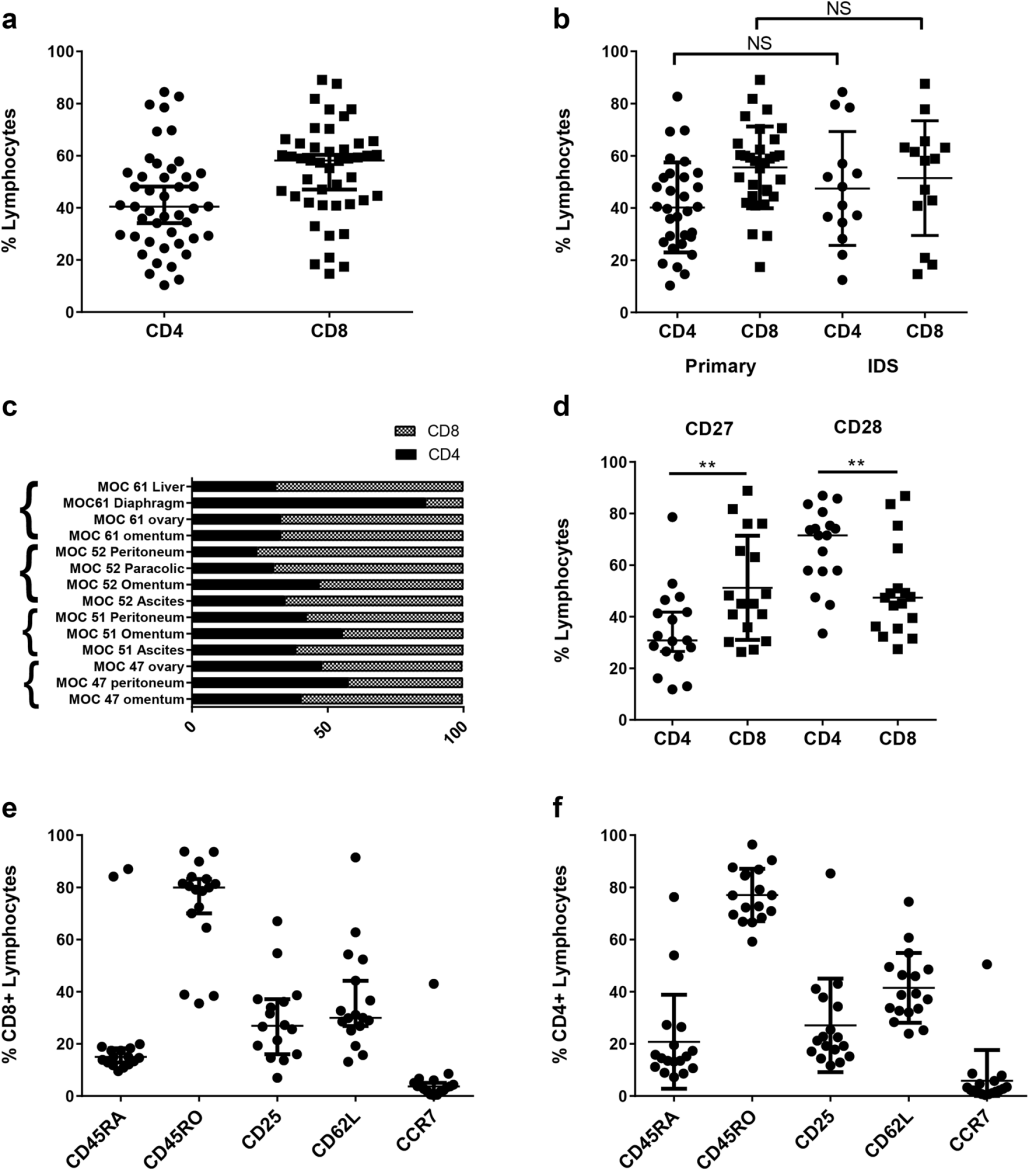
Phenotype of ovarian TIL. Ovarian TILs were stained with CD3, CD4, CD8 and markers associated with T cell differentiation. **a** The proportion of CD4^+^ and CD8^+^ lymphocytes in the expanded TIL populations. **b** Comparison of proportion of CD4^+^ and CD8^+^ lymphocytes in TILs expanded from biopsies taken at primary surgery and following neoadjuvant chemotherapy. **c** Heterogeneity of CD4:CD8 ratio in TILs expanded from biopsies from different anatomical sites. **d** Comparison of CD27 and CD28 expression between CD4^+^ and CD8^+^ T cells. **e** Expression of other T-cell differentiation markers on CD8^+^ and CD4^+^ (**f**) cells. Data are reported as mean % ± SD. **p < 0.01, unpaired Student’s *t* test

Flow cytometry was used to assess the presence or absence of particular immune cells within the expanded cultures. The mean frequency of CD3 expression in the expanded cultures was 96.2% (± 2.8%), confirming that the majority of cells were T lymphocytes. Monocytes (CD14^+^), B cells (CD20^+^) and NK cells (CD56^+^) accounted for < 4% of the cells (Supplementary Fig. 1a). There was no consistent frequency of CD4 and CD8 expression between the expanded cultures. The percentage of CD4^+^ T cells varied between 10.3 and 84.4% and the percentage of CD8^+^ T cells between 14.7 and 89.1% (Fig. 2a). There were no differences in the CD4^+^:CD8^+^ T-cell ratios in TIL cultures expanded from biopsies taken at primary and interval debulking surgery (Fig. 2b). Interestingly, TIL cultures established from tumour biopsies of different anatomical sites from the same patient often showed variable CD4^+^:CD8^+^ T-cell ratios although no consistent pattern was seen (Fig. 2c). Both CD4^+^ and CD8^+^ TILs showed features associated with central-memory (CD27^+^ CCR7^+^ CD62L^−^ CD45RA^−^ CD45RO^+^) and effector-memory (CD27^−^ CCR7^−^ CD62L^−^ CD45RA^−^ CD45RO^+^) phenotypes (Fig. 2d–f). Of note, mean CD28 expression was significantly higher for CD4^+^ T cells (67.14 ± 3.7) compared to CD8^+^ T cells (49.9 ± 4.4) (*p* = 0.004). Conversely, CD27 expression was significantly lower on CD4^+^ T cells (34.7 ± 4.0) compared to CD8^+^ T cells (51.2 ± 4.9) (*p* = 0.009) (Fig. 2d).

### Ovarian TILs have an activated phenotype

**Fig. 3:**
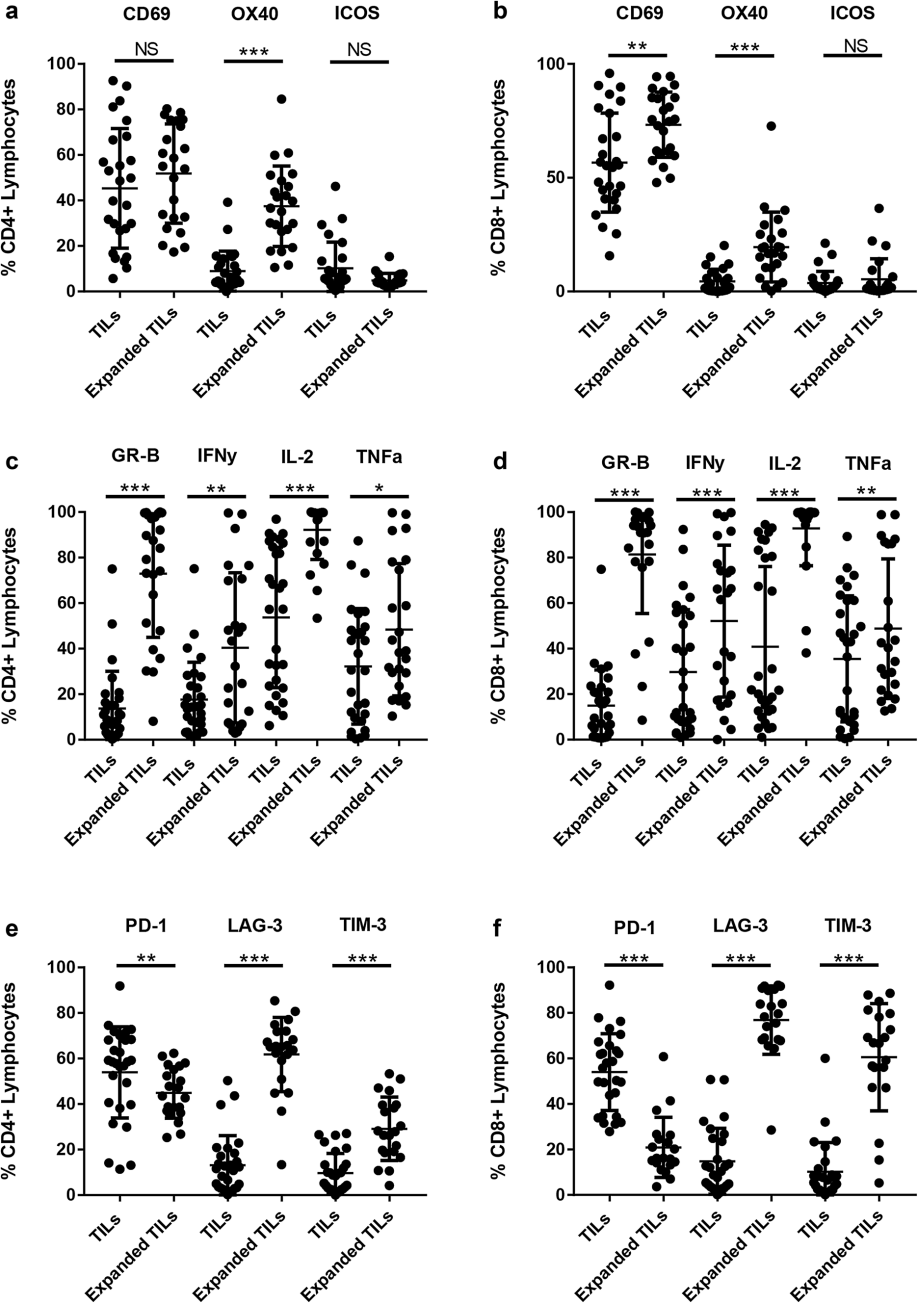
Characterisation of ovarian TILs. Ovarian TILs were characterised phenotypically by flow cytometry at the time of isolation from the tumour digest and following ex vivo expansion. TILs were stained with CD3, CD4, CD8 and markers associated with T cell activation (**a, b**), functional activity (**c, d**), exhaustion (**e, f**). Data are reported as the individual values for each donor and mean ± SD. *p < 0.05, **p < 0.01, ***p < 0.001, paired Student’s *t* test

To determine the effect of ex vivo culture on the phenotype of the ovarian TILs, we measured the expression of markers involved in the activation and function of T cells. Expression of these markers was compared between unexpanded and expanded ovarian TILs. We found that the mean frequency of CD4^+^CD25^Hi^CD127^Lo/Neg^FoxP3^+^ Tregs was low (5.7%, 95% CI 2.7–8.7) in the expanded ovarian TILs as a proportion of the lymphocyte population (Supplementary Fig. 1b). An average of 73.3% of CD8^+^ cells and 51.8% of CD4^+^ cells expressed CD69 indicating the expanded TILs were activated. Our expansion protocol appeared to significantly upregulate OX40 on both the CD4^+^ and CD8^+^ T cells, and CD69 on the CD8^+^ T cells (Fig. 3a, b). Similarly, both CD4^+^ and CD8^+^ expanded TILs had significantly higher expression of functional markers including Granzyme B, IFNγ, IL-2 and TNFα (Fig. 3c, d). More than 50% of CD8^+^ ovarian TILs were positive for IFNγ expression, and more than 75% of CD4^+^ and CD8^+^ TILs were capable of producing Granzyme B.

The co-inhibitory receptor PD-1 has recently been shown to be present on tumour-specific TILs. Here, we found that 53.9% (± 20.0%) of CD4^+^ TILs and 54% (± 16.9%) of CD8^+^ TILs expressed PD-1, at the time of isolation from the digested tumour samples (Fig. 3e, f). Interestingly, the level of PD-1 expression was significantly downregulated following expansion of ovarian TILs. In contrast, TIM-3 and LAG-3 expression were upregulated in the expanded TILs, particularly in CD8^+^ TIL.

### Expanded TILs retain functional activity

**Fig. 4:**
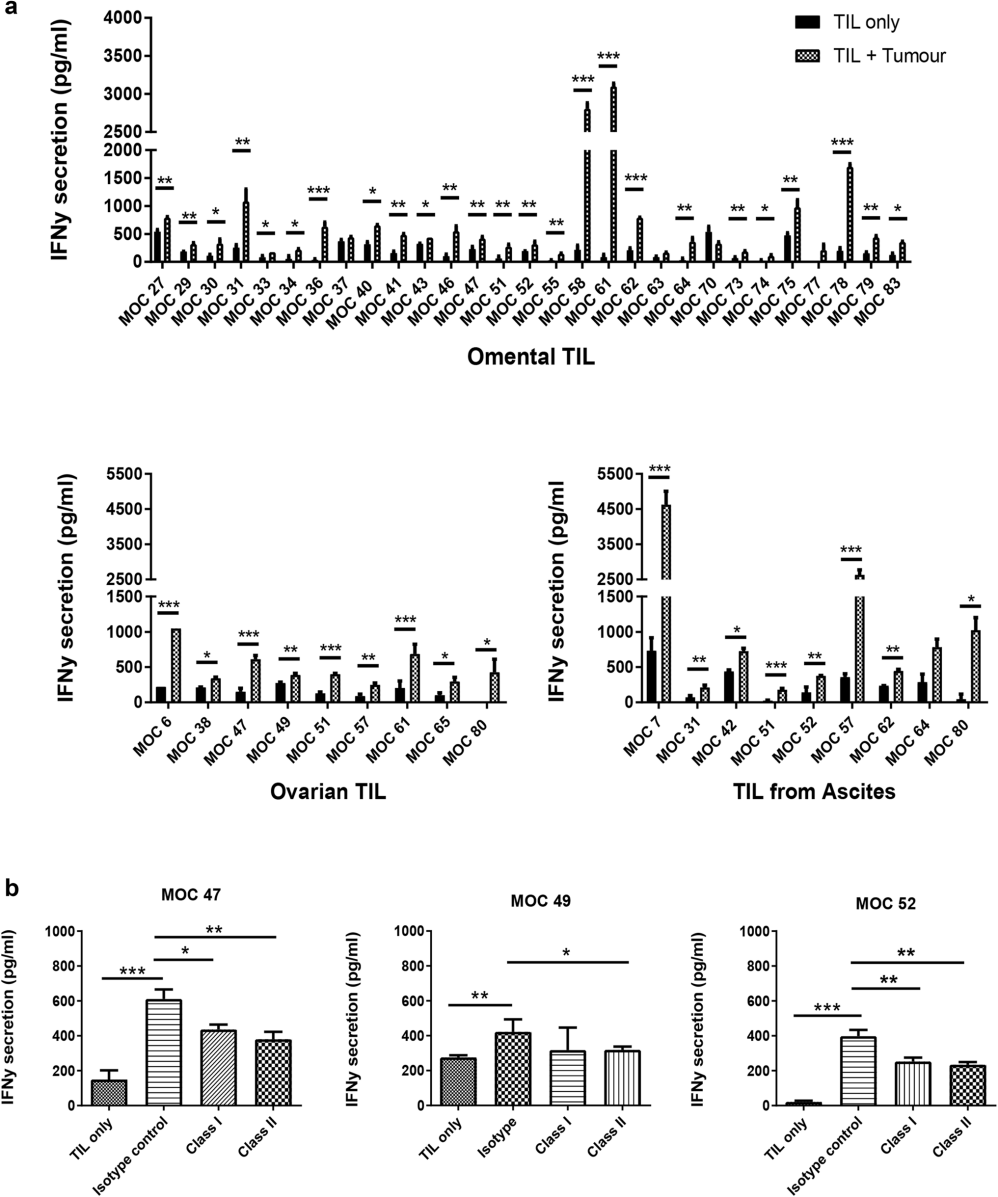
Functional activity of expanded ovarian TILs against autologous tumour cells. **a** IFNγ release after 24-h co-culture of TILs with autologous tumour. TILs were considered to show functional activity if significantly higher levels of IFNγ were produced by the co-culture compared to TILs alone. Mean and SD of three replicates are shown. *p < 0.05, **p < 0.01, ***p < 0.001, paired Student’s *t* test. **b** Effect of MHC class I and II blockade on TIL functional activity against autologous tumour. Expanded TILs were co-cultured against autologous tumour cells that have been pre-incubated with either an anti-MHC class I or anti-MHC class II antibody, and IFNγ release compared. Representative results from three donors are shown. Mean and SD of three replicates are shown. *p < 0.05, **p < 0.01, ***p < 0.001; one-way ANOVA with multiple comparisons

We sought to determine whether expanded TILs retained the ability to recognize and respond to autologous tumour cells in vitro. We assessed this by measuring IFNγ release by TILs upon encountering cognate antigen. Where available, TILs were co-cultured against autologous tumour cells (either cryopreserved from the tumour disaggregate or cultured from the tumour) at a 1:1 ratio. TILs were concurrently co-cultured with PMA and Ionomycin as a positive control, to confirm that the TILs were able to produce high levels of cytokine. Figure 4a demonstrates that 94% of autologous co-cultures demonstrated IFNγ secretion significantly above that of TILs alone. Of note, in four patients where TILs were successfully expanded from different anatomical sites, all TIL cultures were reactive against autologous tumour from the cognate site (supplementary Fig. 2). We found no significant difference in the amount of IFNγ produced by expanded T cells between the different molecular subtypes of ovarian cancer as determined by Nanostring (supplementary Fig. 3b). To determine the T-cell restriction of functional activity, autologous tumour cells were pre-incubated with anti-MHC class I- and anti-MHC class II-specific antibodies, prior to setting up the co-culture assays (Fig. 4b). Interestingly, both class I- and class II-specific blockade consistently reduced functional activity of TIL, suggesting that both CD4^+^ and CD8^+^ TILs contribute to functional activity of the expanded TILs against autologous tumour.

**Fig. 5:**
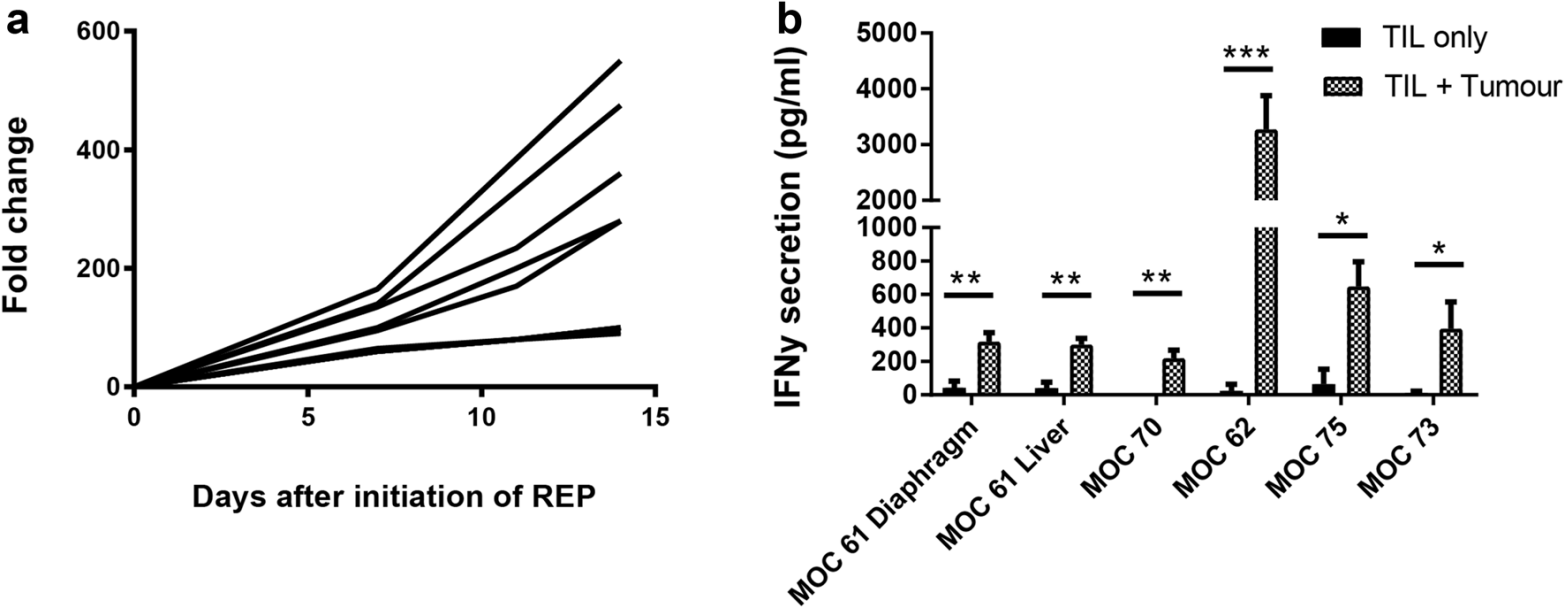
Expansion and functional activity of TILs following rapid expansion protocol. **a** Fold change of nine TIL cultures during rapid expansion (REP). **b** IFNγ release after 24-h co-culture of rapidly-expanded TILs with autologous tumour. Data represent mean ± SD. *p < 0.05, **p < 0.01, ***p < 0.001, paired Student’s *t* test

A subset of TIL cultures that had been cryopreserved following initial expansion was thawed and subjected to a small-scale REP to determine whether TILs could be expanded to clinically relevant numbers. Nine TIL cultures were chosen—three with an initial yield of > 1 × 10^8^, three with an initial yield of 1.5 × 10^7^–1 × 10^8^ and three with an initial yield of < 1.5 × 10^7^. Rapid expansion for 14 days resulted in 229.9 ± 179.0-fold expansion (Fig. 5a). Autologous tumour was available for six of these patients. Figure 5b shows that expanded TILs from these six donors maintained functional activity against autologous tumour following REP.

## Discussion

TIL therapy has emerged as a unique opportunity to exploit the specificity and diversity of a patient’s immune response. The T cells present within the TME represent a polyclonal repertoire of TCRs specific for a vast array of defined and undefined epitopes. Given the heterogeneity observed in epithelial ovarian cancer, utilization of such an individualized immunotherapy may have a significant impact on patient survival. TIL therapy has been successfully adopted for metastatic melanoma [22, 37–40]. Although previous clinical trials investigating TIL therapy in other solid tumours have shown only modest benefit at best, recent developments in TIL generation strategies have reinvigorated interest in developing this approach in other immunogenic cancers. In particular, new insights into the effect of prolonged culturing techniques on T-cell differentiation and function, and the development of optimal co-stimulation conditions that allow preferential expansion of specific T-cell subsets, have revolutionized this field. Strategies employed for ex vivo culture of ovarian TIL in the four early trials included high-dose IL-2 alone [27, 28, 31] or in combination with anti-CD3 stimulation [29, 30]. Interestingly, the best clinical results yielded from the trial that combined anti-CD3 activation and IL-2. It is worth noting that these trials generally enrolled patients with recurrent or refractory disease, where immunosuppression is likely to be a dominant feature.

In the present study, we explored the feasibility of expanding TILs from epithelial ovarian cancer biopsies. Using anti-CD3/anti-CD28 Dynabeads and high-dose IL-2, TILs were expanded from 89.7% of surgically resected ovarian tumours. TIL yields were similar to those previously reported for melanoma and renal cell carcinoma [23, 24, 41]. Interestingly, we were only able to expand TILs from one of the three patients with recurrent disease. This may have been due to a paucity of TILs in these biopsies or T cell anergy, but requires a larger cohort to investigate further. In the five patients where TILs were expanded from biopsies of disease from different anatomical sites, omental TILs consistently expanded more readily than other anatomical sites. It has been shown in mouse models that B and T lymphocytes in the peritoneal cavity traffic to the omentum [42]. Further studies looking at the density and distribution of TIL and other immune cells within the omentum may provide an explanation for this.

Four molecular subtypes of high-grade serous ovarian cancer (C1/mesenchymal, C2/immune, C4/differentiated, C5/proliferative) have previously been described by Tothill et al. [35]. Tumours with a C2 or C4 subtype are characterized by high infiltration of T cells into the tumour islets and the surrounding stroma. As such, we hypothesized that TILs would expand more readily from patients with a C2 or C4 subtype. Somewhat surprisingly, we found that there was no correlation between the molecular subtypes of HGSC and the efficiency of TILs expansion suggesting that other stratification techniques are likely to be required. Interestingly, all of the subtypes with the exception of the C5 subtype had either high numbers of CD3^+^ lymphocytes in the tumour and/or stromal compartments in the Tothill characterization. When initiating the TIL cultures, it is likely that the disaggregated tumour cells contain both stromal and intratumoral lymphocytes, and this may in part, explain the lack of correlation between molecular subtypes and efficiency of TIL expansion. In our cohort, there was a tendency to lower numbers of CD3 T cells in the C5 subtype; however, there were not a sufficient number of tumours in this subgroup to demonstrate significance (supplementary Fig. 3a). Despite this, the T cells expanded as well as the other molecular subtypes. Importantly, we have demonstrated marked expansion of TIL in vitro even in the presence of low CD3 + infiltration in the original tumour digests (supplementary Fig. 3b). This suggests that the use of anti-CD3/anti-CD28 beads in our protocol provides sufficient stimulation to enable expansion of TILs from tumours with low CD3^+^ infiltration.

It has long been established that T cells within the tumour microenvironment are functionally suppressed. CD8^+^ TILs have been shown to express higher levels of PD-1 than CD8^+^ T cells isolated from peripheral blood, and CD8^+^PD-1^+^ TILs have impaired effector function with significantly reduced cytokine production [43]. In the current study, expanded ovarian TILs were predominantly activated, with high expression of CD69 and low expression of PD-1. Expanded TILs also demonstrated significantly higher levels of intracellular cytokines compared to TILs isolated from the tumour digests. These results demonstrate the fluidity of the ovarian TIL phenotype and suggest that by expanding TILs away from the inhibitory tumour microenvironment, the scale can be tipped back in favour of tumour elimination. Importantly, the use of high-dose IL-2 in addition to paramagnetic beads did not polarize ovarian TIL cultures towards a regulatory T-cell phenotype.

Interestingly, we observed increased expression of TIM-3 and LAG-3 in the expanded TILs. These molecules are generally considered to be co-inhibitory receptors and upregulated in response to activation and differentiation [44, 45]. There have been conflicting reports in the literature, with regard to the functional and phenotypic implications of T cells that express PD-1, TIM-3 or LAG-3. A recent study by Gros et al. [46] demonstrated that expression of PD-1, TIM-3 or LAG-3 on CD8^+^ TILs identifies the tumour-reactive clonotypes, and that PD-1^+^, TIM-3^+^ and LAG-3^+^ CD8^+^ melanoma TILs expanded in IL-2 were capable of secreting IFNγ and lysing tumour in vitro. Similarly, it has also been reported that TIM-3 is preferentially expressed on IFNγ-secreting cells [47]. Importantly, our findings are in agreement with previous reports, which have shown increased TIM-3 and LAG-3 expression and decreased PD-1 after expansion [46, 48]. It is well known that expanding TILs in the presence of cytokines such as IL-2 and anti-CD3/anti-CD28 can change the expression of inhibitory receptors in addition to the activation markers on T cells [48]. Despite high expression of these markers on the expanded TILs, TILs showed significant functional activity in response to autologous tumour.

Previous clinical trials of TIL therapy have assessed the efficacy of adoptively transferred populations of effector T cells [49]. More recently, the results of several in vivo studies have shown that less differentiated T cells possess enhanced engraftment [50]. In this study, ovarian TILs showed features associated with the less differentiated central memory (CD27^+^ CCR7^+^ CD62L^−^ CD45RA^−^ CD45RO^+^) and effector memory (CD27^−^ CCR7^−^ CD62L^−^ CD45RA^−^ CD45RO^+^). It was difficult to classify the differentiation status of expanded TILs into distinct categories, as although CCR7 expression was low signifying effector-memory cells, CD27 and CD62L expression were moderately high indicating that these cells may be of a central-memory phenotype. Stimulation of the TILs by anti-CD3/anti-CD28 beads and high-dose IL-2 may account for this.

To test the activity of the ovarian TILs against tumour antigens, we co-cultured the TILs with autologous tumour cells and measured IFNγ release. Our results indicate that > 90% of the established TIL cultures were functionally reactive. This is considerably higher than previously published in other pre-clinical studies [23, 24, 41, 51]. Importantly, we used the combined mechanical/enzymatic GentleMACS system to disaggregate the tumours, which has been shown to preserve cell surface antigen expression better than standard enzymatic disaggregation [23]. As such, the target cells utilized in our assay may be more consistently recognized by the expanded TILs.

Flow cytometry analysis revealed that expanded TIL cultures demonstrated highly heterogeneous ratios of CD4^+^:CD8^+^ T cells. Dudley et al. [36] reported that most melanoma-derived TIL cultures were predominantly CD8^+^; however, there was a significant proportion of CD4^+^ T cells observed in the ovarian TIL cultures. Similar proportions of CD4^+^ T cells have been reported in TILs derived from renal cell carcinoma specimens [23, 24], and suggest that CD4^+^ cells may too have a functional role. Indeed, anti-tumour activity was consistently reduced when TILs were co-cultured with autologous tumour cells in the presence of an anti-MHC class II antibody affirming that CD4^+^ TILs contribute to functional activity.

In summary, our results indicate that TILs can be readily isolated and expanded from epithelial ovarian cancer, in both the primary and neo-adjuvant setting. These TILs are characterized by a more activated and functional phenotype than the TILs present in the initial tumour digest. What’s more, expanded TILs retained the ability to recognize autologous tumour cells in vitro. Based on our results, we are currently developing an early-phase clinical trial protocol to evaluate the clinical potency of TILs in ovarian cancer.

## Electronic supplementary material

Below is the link to the electronic supplementary material.

Supplementary material 1 (PDF 691 KB)

## Abbreviations

EOC: Epithelial ovarian cancer
IDS: Interval debulking surgery
NICE: National Institute for Health and Care Excellence
REP: Rapid expansion protocol
