# Supplementary material for: Ex vivo expanded tumour-infiltrating lymphocytes from ovarian cancer patients release anti-tumour cytokines in response to autologous primary ovarian cancer cells
Source: Cancer Immunol Immunother. 2018 Jul 23;67(10):1519–31. doi: 10.1007/s00262-018-2211-3 (PMC6182400; doi:10.1007/s00262-018-2211-3)
Supplement: Supplementary file 1 — Supplementary material 1 (PDF 691 KB) [file 262_2018_2211_MOESM1_ESM.pdf]

Supplementary data

Supplementary Table 1. Flow cytometry antibodies

Name	Fluorophore	Clone	Company	Panel used
**Anti-CCR7**	APC	REA108	Miltenyi, Germany	Differentiation
**Anti-CD45RA**	PE	HI100	BD Biosciences, UK	
**Anti-CD45RO**	PE	UCHL1		
**Anti-CD28**	PE	CD28.2		
**Anti-CD27**	PE	M-T271		
**Anti-CD25**	PE	M-A251		
**Anti-CD8**	FITC	HIT8A		
**Anti-CD62L**	Pe/Cy7	DREG-56	eBioscience, UK	
**Live/Dead**	BV395	N/A	Invitrogen	Activation, Treg, Exhaustion & Functional Panels
**Anti-CD4**	BV785	OKT4	Biolegend, UK	
**Anti-CD14**	Pe-Cy7	HCD14		
**Anti-CD20**	Pe-Cy7	2H7		
**Anti-CD56**	Pe-Cy7	HCD56		
**Anti-CD45**	BV510	UCHL1		
**Anti-CD3**	APC	UCHT1		
**Anti-PD-1**	PE	EH12.2H 7		
**Anti-TIM3**	BV421	F38-2E2		
**Anti-CD69**	BV421	FN50		
**Anti-CD25**	BV711	BC96		
**Anti-OX40**	FITC	ACT35		
**Anti-Foxp3***	PE	206D		
**Anti-CD127**	BV421	A019D5		
**Anti- TNFα***	BV650	MAb11		
**Anti- IFNγ***	BV711	4S.B3		
**GranzymeB**	FITC	GB11		
**Anti-ICOS**	BV650	Dx29	BD Biosciences, UK	
**Anti-CD8**	APC-H7	SK1		
**Anti-IL2***	BV421	5344.111		
**Anti-LAG3**	FITC	17B4	Enzo Lifesciences	

*Denotes intracellular antigens

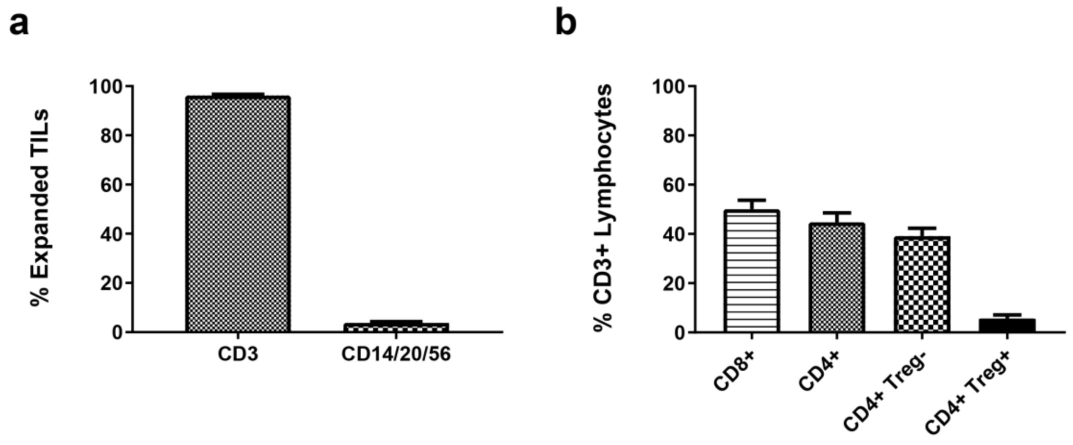

Supplementary Figure 1. Phenotype of expanded ovarian TILs. (a) Frequency of $CD3^{+}$ T cells and other lineages ($CD14^{+}$/$CD20^{+}$/$CD56^{+}$) in the expanded TIL cultures. (b) Percentage $CD8^{+}$, $CD4^{+}$ and Treg cells in the expanded TILs cultures. Data are reported as mean % ± SEM.

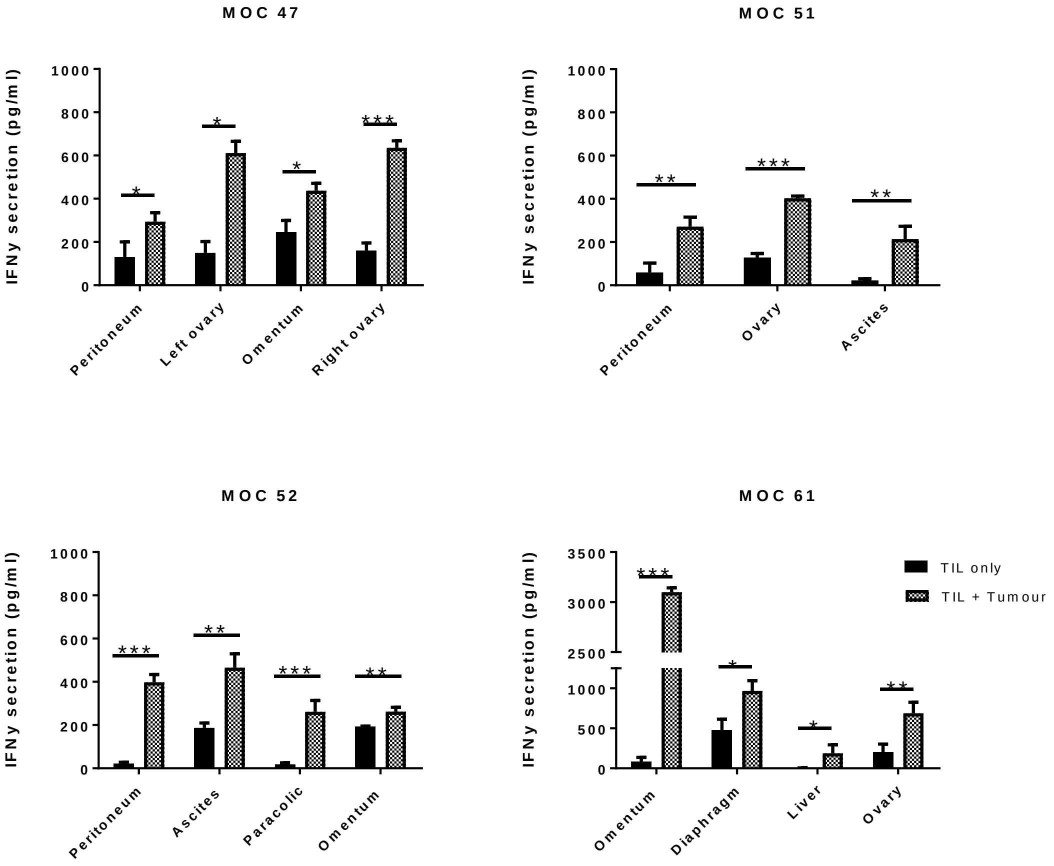

Supplementary Figure 2. Comparison of functional activity of TILs expanded from different anatomical sites. In four patients, TILs were successfully expanded from a minimum of three different anatomical sites. Expanded TILs were co-cultured against autologous tumour from cognate sites, to determine whether expanded TILs retained functional activity. TILs were considered to show functional activity if significantly higher levels of IFNγ were produced by the co-culture compared to TILs alone. Mean and SD of three replicates are shown. *P<0.05, **P<0.01, ***P<0.001, paired student T-test.

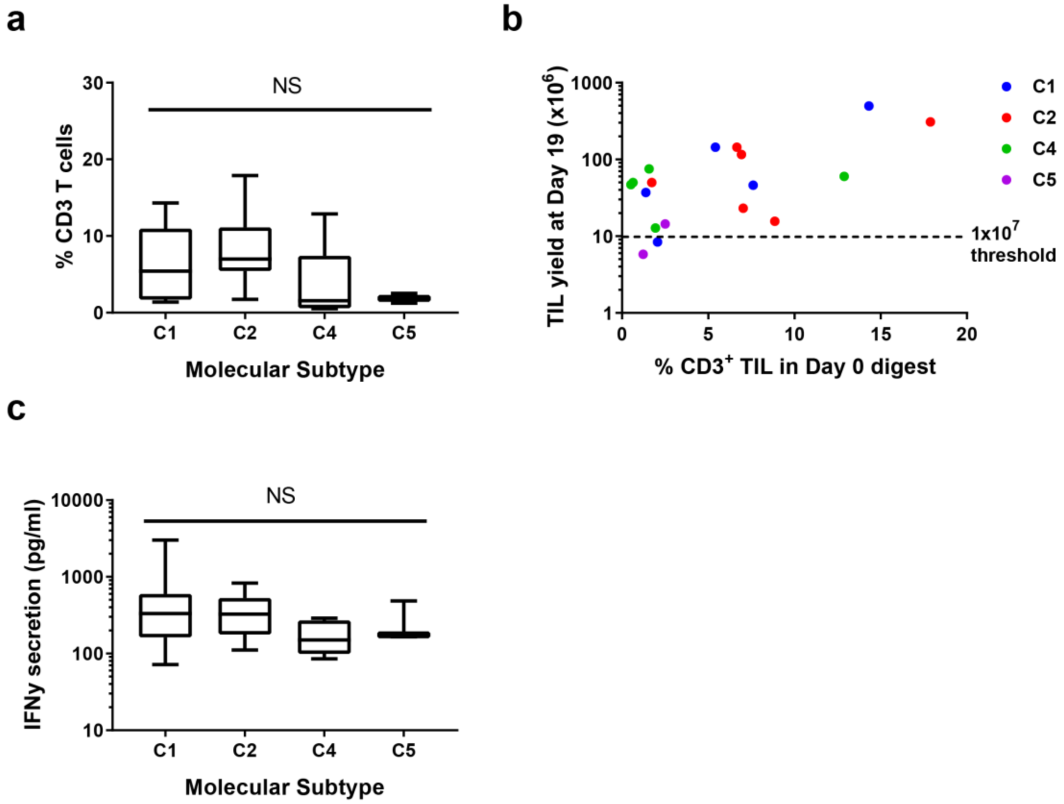

Supplementary Figure 3. Comparison of CD3 T cell infiltration, expansion and IFNγ secretion by the different molecular subtypes of ovarian cancer. (a) Percentage of CD3+ T cells in the tumour digest was determined using flow cytometry and compared between molecular subtypes of ovarian cancer (C1/mesenchymal; C2/immune; C4/differentiated; C5/proliferative). (b) Final TIL yield at day 19 compared against percentage of CD3+ TIL in tumour digest. Dashed line illustrates $1x10^7$ threshold for a positive TIL culture. (c)IFNγ produced by expanded TILs in response to autologous tumour cells was compared between molecular subtypes. Data represents the median, upper and lower quartiles and range. Kruskal-Wallis test; P<0.05, NS, not significant.
